# Therapeutic potential of heterocyclic pyrimidine scaffolds

**DOI:** 10.1186/s13065-018-0406-5

**Published:** 2018-04-04

**Authors:** Sanjiv Kumar, Balasubramanian Narasimhan

**Affiliations:** Faculty of Pharmaceutical Sciences, Maharshi Dayanand University, Rohtak, 124001 India

**Keywords:** Pyrimidine derivatives, Antimicrobial, Antioxidant, Antimalarial, Anticancer, Anti-inflammatory

## Abstract

Heterocyclic compounds offer a high degree of structural diversity and have proven to be broadly and economically useful as therapeutic agents. Comprehensive research on diverse therapeutic potentials of heterocycles compounds has confirmed their immense significance in the pathophysiology of diseases. Heterocyclic pyrimidine nucleus, which is an essential base component of the genetic material of deoxyribonucleic acid, demonstrated various biological activities. The present review article aims to review the work reported on therapeutic potentials of pyrimidine scaffolds which are valuable for medical applications during new generation.
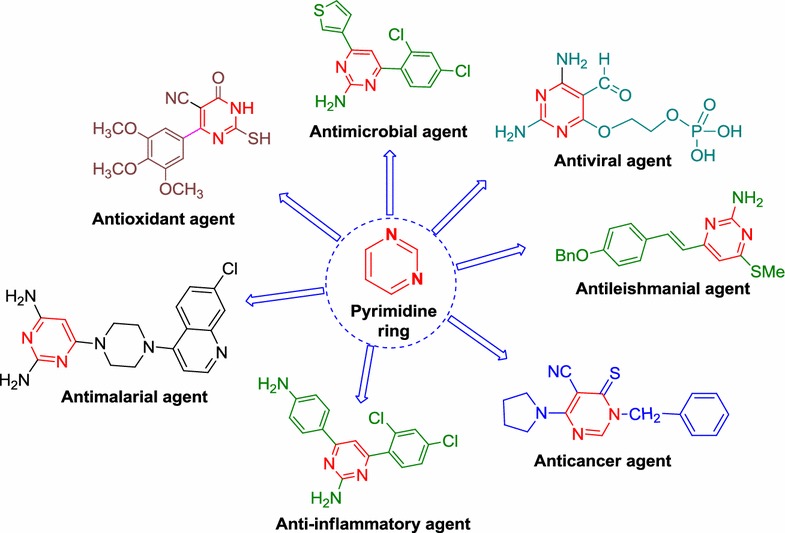

## Introduction

**Fig. 1:**
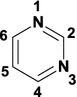
Pyrimidine ring

Pyrimidine is the six membered heterocyclic organic colorless compound containing two nitrogen atoms at 1st and 3rd positions (Fig. 1). The name of the pyrimidine was first applied by Pinner from the combination of two words pyridine and amidine). Pyrimidines(1,3-diazines) and their fused analogues form a large group of heterocyclic compounds. Pyrimidine which is an integral part of DNA and RNA imparts diverse pharmacological properties. The pyrimidine have been isolated from the nucleic acid hydrolyses and much weaker base than pyridine and soluble in water [1]. Pyrimidine and its derivatives have been described with a wide range of biological potential i.e. anticancer [2], antiviral [3], antimicrobial [4], anti-inflammatory [5], analgesic [6], antioxidant [7] and antimalarial [8] etc.

## Biological significance of pyrimidine scaffolds

### Antimicrobial activity

The growing health problems demands for a search and synthesis of a new class of antimicrobial molecules which are effective against pathogenic microorganisms. Despite advances in antibacterial and antifungal therapies, many problems remain to be solved for most antimicrobial drugs available. The extensive use of antibiotics has led to the appearance of multidrug resistant microbial pathogens which necessitated the search for new chemical entities for treatment of microbial infections [9].

**Fig. 2:**
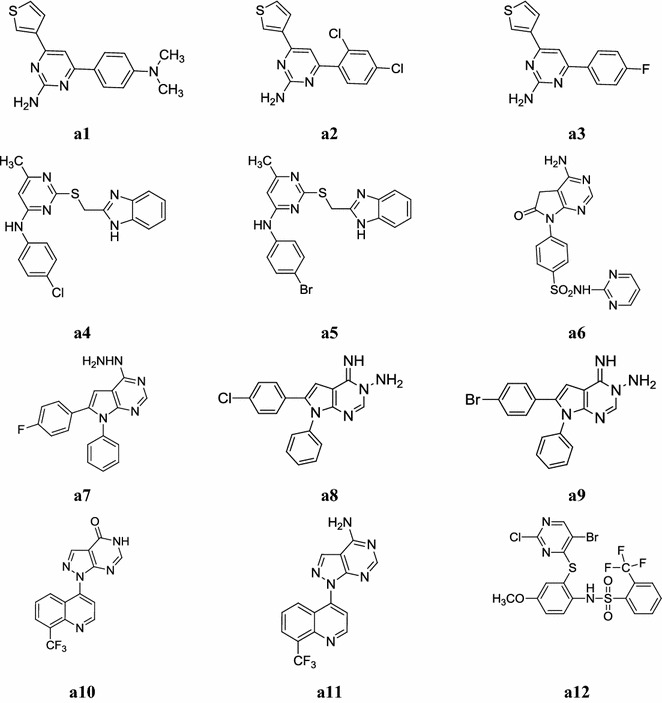
Chemical structure of the most active antimicrobial pyrimidine derivatives (**a1**–**a12**)

Anupama et al. synthesized a series of 2,4,6-trisubstituted pyrimidines by reacting chalcone with guanidine hydrochloride. All the synthesized derivatives were confirmed by physicochemical properties and spectral data (IR, NMR and elemental analyses) and screened their in vitro antimicrobial activity against bacterial and fungal strains by cup plate method using Mueller–Hinton agar medium. Among the derivatives tested, compounds, **a1**, **a2** and **a3** exhibited promising activity against microbial strains (*B. pumilis, B. subtilis, E. coli, P. vulgaris. A. niger* and *P. crysogenium*) and showed activity comparable with standard drugs. Structure activity relationship (SAR) studies indicated that compounds, **a1**, **a2** and **a3** having dimethylamino, dichlorophenyl and fluorine substituent on the phenyl ring at 4th position respectively exhibited better antimicrobial activity (Table 1, Fig. 2) [4].

**Table 1:** Antimicrobial activity of compounds **(a1**–**a3)**

Compounds	Zone of inhibition (in mm)					
	Microbial species					
	*B. subtilis*	*B. pumilis*	*E. coli*	*P. vulgaris*	*A. niger*	*P. crysogenium*
**a1**						
A	15	12	11	12	11	12
B	20	14	20	18	13	14
**a2**						
A	16	13	12	15	16	15
B	20	15	21	21	18	18
**a3**						
A	17	14	13	14	15	14
B	20	15	21	20	17	17
C	–	–	–	–	–	–
S						
A	25	29	26	28	23	24
B	30	31	29	31	28	27

A: 0.05 ml (50 μg); B: 0.1 ml (100 μg); C: control (DMSO); S: standard (benzyl penicillin for bacterial strains) and fluconazole for fungal strains

Chen et al. synthesized a novel series of 4-substituted-2-{[(1*H*-benzo[*d*]imidazol-2-yl) methyl]thio}-6-methylpyrimidines from pyrimidine–benzimidazole combination. All the synthesized derivatives were fully characterized by ^1^H-NMR, ^13^C-NMR and HRMS study and screened its in vitro antimicrobial activity against Gram-positive bacteria (*Staphylococcus aureus, Bacillus subtilis*), Gram-negative bacteria (*Escherichia coli, Stenotrophomonas maltophilia*) and fungi (*Candida albicans*). The minimum inhibitory concentration (MIC) of the target compounds was determined by broth microdilution method and compared to two commercial antibiotics (levofloxacin and fluconazole). Among the entire synthesized derivatives, compounds, **a4** and **a5** were found to be the most active antimicrobial agents (Table 2, Fig. 2). Structure activity relationship showed that aromatic amines at pyrimidine ring are beneficial for the antimicrobial activity. Besides, the aniline containing *para*-substituted groups (especially Cl and Br) is more beneficial for the activity [10].

**Table 2:** Antimicrobial activity (MIC = µg/ml) of compounds **a4** and **a5**

Compounds	Bacterial strains				Fungal strain
	*Staphylococcus aureus*	*Bacillus subtilis*	*Escherichia coli*	*Stenotrophomonas maltophilia*	*Candida albicans*
**a4**	8	128	128	2	64
**a5**	16	128	128	4	8
Levofloxacin	0.5	0.25	0.125	0.25	–
Fluconazole	–	–	–	–	2

El-Gaby et al. developed a new class of pyrrolo[2,3-*d*]pyrimidines containing sulfonamide moieties and screened its in vitro antifungal activity against four species of fungi viz: *Aspergillus ochraceus* (Wilhelm), *Penicillium chrysogenum* (Thom), *Aspergillus fleavus* (Link) and *Candida albicans* (Robin) Berkho by disc diffusion technique. Most of the synthesized molecules in this series were found to possess antifungal activity (Table 3, Fig. 2) towards all the microorganisms’ used especially, compound **a6** exhibited a remarkable antifungal activity which is comparable to the standard fungicide drug mycostatin [11].

**Table 3:** Antifungal activity of synthesized compound **a6**

Compound	Zone of inhibition (mm)			
	Fungal species			
	*A*. *ochraceus* (AUCC-230)	*P*. *chrysogenum* (AUCC-530)	*A*. *flea*v*us* (AUCC-164)	*C*. *albicans* (AUCC-1720)
**a6**	18 (45%)	14 (37%)	16 (42%)	34 (85%)
Mycostatine	40 (100)	38 (100%)	38 (100%)	40 (100%)

Hilmy et al. developed a new series of pyrrolo[2,3-*d*]pyrimidine derivatives. The synthesized compounds were confirmed by IR, NMR, Mass and elemental analysis study and evaluated its antimicrobial activity against bacterial (*Staphylococcus aureus*, *Escherichia coli*) and fungal (*Candida albicans*) organisms was carried out by serial dilution method. All synthesized derivatives showed that good antimicrobial activity, especially, compounds, **a7**, **a8**, **a9** were exhibited the better antimicrobial activity and compared with the standard drug (ampicillin and fluconazole) (Table 4, Fig. 2) [12].

**Table 4:** The MIC (mg/ml) value of the compounds **a7**, **a8** and **a9** tested against organisms

Compounds	Antimicrobial results (MIC = mg/ml)		
	*Escherichia coli*	*Staphylococcus aureus*	*Candida albicans*
**a7**	1.25	0.31	0.31
**a8**	1.25	0.31	0.62
**a9**	1.25	0.31	0.31
Ampicillin	1.25	0.62	–
Fluconazole	–	–	1.5

Holla et al. developed a new class of pyrazolo[3,4-*d*]pyrimidine derivatives. The synthesized derivatives were analyzed for N content and their structures were confirmed by IR, NMR and Mass spectral data and screened their antibacterial activity against *Escherichia coli*, *Staphylococcus aureus*, *Pseudomonas aeruginosa* and *Bacillus subtilis* by disk diffusion method and antifungal activity against *Aspergillus flavus*, *Aspergillus fumigates, Candida albicans*, *Penicillium marneffei* and *Trichophyton mentagrophytes* by serial plate dilution method. All synthesized pyrazolo[3,4-*d*]pyrimidine derivatives in this series showed that good antimicrobial and fungal activity against bacterial and fungal strains, especially compounds, **a10** displayed very good antibacterial activity (Table 5, Fig. 2) and **a11** exhibited antifungal activity (Table 6, Fig. 2) [13].

**Table 5:** Antibacterial activity data of compound **a10**

Compound	Zone of inhibition (mm) of bacterial species			
	*Escherichia coli*	*Staphylococcus aureus*	*Pseudomonas aeruginosa*	*Bacillus subtilis* (recultured)
**a10**	28	25	24	26
Streptomycin	20	21	24	24

**Table 6:** Antifungal activity data of prepared compound **a11**

Compound	Zone of inhibition (mm) of fungal species		
	*Aspergillus flavus*	*Aspergillus fumigatus*	*Trichophyton mentagrophytes* (recultured)
**a11**	25	22	24
Fluconazole	21	18	19

Mallikarjunaswamy et al. synthesized a series of novel 2-(5-bromo-2-chloro-pyrimidin-4-ylsulfanyl)-4-methoxy-phenylamine derivatives by the reaction of 2-(5-bromo-2-chloro-pyrimidin-4-ylsulfanyl)-4-methoxy-phenylamine with various sulfonyl chlorides and its molecular structures were characterized by elemental analyses, FT-IR, ^1^H-NMR and LC–MS spectral studies and screened in vitro antimicrobial activity against Gram-positive bacteria (*Bacillus subtilis*, *Staphylococcus aureus*) and Gram-negative bacteria (*Xanthomonas campestris* and *Escherichia coli*) in dimethylformamide by disc diffusion method on nutrient agar medium and antifungal activity against *Fusarium oxysporum* in dimethylformamide by poisoned food technique. Among them, compound **a12** was found to be most potent against fungal strain (*Fusarium oxysporum*) and bacterial strains (*Bacillus subtilis*, *Staphylococcus aureus*, *Xanthomonas campestris* and *Escherichia coli*) and compared with standard antimicrobial drugs (Table 7, Fig. 2) [9].

**Table 7:** In vitro antibacterial and antifungal activities of compound **a12**

Compound	Zone of inhibition in diameter (mm) % inhibition				
	Microbial species				
	*B. subtilis*	*S. aureus*	*X. campestris*	*E. coli*	*F. oxysporum*
**a12**	33	29	32	33	96.9
Bacteriomycin	–	–	34	–	–
Gentamycin	35	30	–	35	–
Nystatin					100

**Fig. 3:**
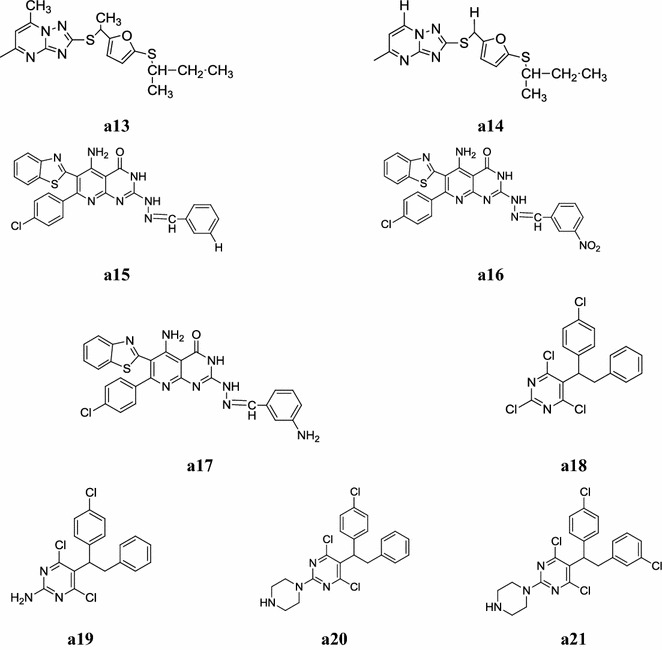
Chemical structure of the most active antimicrobial pyrimidine derivatives (**a13**–**a21**)

A new series of 1,2,4-triazolo[1,5-*a*]pyrimidine derivatives bearing 1,3,4-oxadiazole moieties was designed and synthesized by Chen et al. The molecular structures of all new compounds were characterized by spectral means (^1^H-NMR, Mass and elemental analyses) and evaluated their in vitro antifungal activity against *Rhizoctonia solani*. In this series, compounds, **a13** and **a14** displayed the highest antifungal activity against *Rhizoctonia solani* with EC_50_ = 3.34 µg/ml and EC_50_ = 6.57 µg/ml values respectively than the carbendazim (EC_50_ = 7.62 µg/ml) due to presence of the *sec*-butyl group (Fig. 3) [14].

A new library of 5-amino-6-(benzo[*d*]thiazol-2-yl)-2-(2-(substituted benzylidene) hydrazinyl)-7-(4-chlorophenyl)pyrido[2,3-*d*]pyrimidin-4(3*H*)-one derivatives was synthesized by Maddila et al. and evaluated its antibacterial activity against *Staphylococcus aureus, Escherichia coli, Klebsiella pneumoniae, Pseudomonas aeruginosa* and *Streptococcus pyogenes* and antifungal activity against *Aspergillus flavus, Aspergillus fumigatus, Candida albicans, Penicillium marneffei* and *Mucor* by the twofold serial dilution method. Compounds, **a15**, **a16** and **a17** showed excellent antibacterial and antifungal activity than the standard drugs ciprofloxacin and clotrimazole respectively (Tables 8, 9, Fig. 3) [15].

**Table 8:** Antibacterial activity results of compounds **(a15**–**a17)**

Compounds	Minimum inhibitory concentration (MIC = µg/ml)				
	Bacterial species				
	*S. aureus*	*E. coli*	*K. pneumoniae*	*P. aeruginosa*	*S. pyogenes*
**a15**	12.5	25	25	25	12.5
**a16**	12.5	12.5	12.5	12.5	12.5
**a17**	25	12.5	12.5	25	12.5
Ciprofloxacin	25	25	50	25	12.5

**Table 9:** Antifungal activity results of compounds **(a15**–**a17)**

Compounds	Minimum inhibitory concentration (MIC = µg/ml)				
	Fungal species				
	*A. flavus*	*A. fumigatus*	*C. albicans*	*P. marneffei*	*Mucor*
**a15**	12.5	12.5	25	25	12.5
**a16**	12.5	12.5	12.5	12.5	12.5
**a17**	25	12.5	25	12.5	25
Clotrimazole	25	25	50	25	50

Fellahil et al. synthesized a new series of 5-(1,2-diarylethyl)-2,4,6-trichloro pyrimidines and 2-amino- and 2-(1-piperazinyl)-5-(1,2-diarylethyl)-4,6-dichloro pyrimidines via organozinc reagents and demonstrated its antibacterial activity against human bacterial flora. Biological tests showed that 5-[1-(4-chlorophenyl)-2-phenylethyl]-2,4,6-trichloro pyrimidine derivatives i.e. compounds **a18** and **a19** were found to be most active against wide range of bacterial flora of the axilla and foot, while 2-(1-piperazinyl)-4,6-dichloro pyrimidine derivatives **a20** and **a21** displayed a great selectivity against *Corynebacterium xerosis* and *Arcanobacterium haemolyticum* of the human axilla (Table 10, Fig. 3) [16].

**Table 10:** Pharmacological evaluation (MIC = µg/ml) of the 2-substituted 5-(1,2-diarylethyl)-4,6-dichloropyrimidines

	**a18**	**a19**	**a20**	**a21**
Axillary bacterial flora				
*Staphylococcus xylosus*	20	100	100	100
*Staphylococcus epidermidis*	100	100	100	75
*Staphylococcus haemolyticus*	100	100	100	50
*Corynebacterium xerosis*	20	30	30	30
*Micrococcus luteus*	20	100	100	100
*Arcanobacterium haemolyticum*	10	10	10	10
Foot bacterial flora				
*Staphylococcus epidermidis*	> 100	100	100	75
*Staphylococcus hominis*	100	100	100	75
*Staphylococcus cohnii*	100	100	100	75
*Corynebacterium* sp. g C	100	100	100	75
*Corynebacterium* sp. g B	30	100	100	50
*Corynebacterium* sp. g D2	30	100	50	50
*Micrococcus luteus*	20	100	100	75
*Micrococcus sedentarius*	30	100	100	75
*Acinetobacter* sp.	> 1000	> 500	50	30
*Moraxella* sp.	300	30	100	50
*Alcaligenes* sp.	1000	> 500	> 500	> 500

**Fig. 4:**
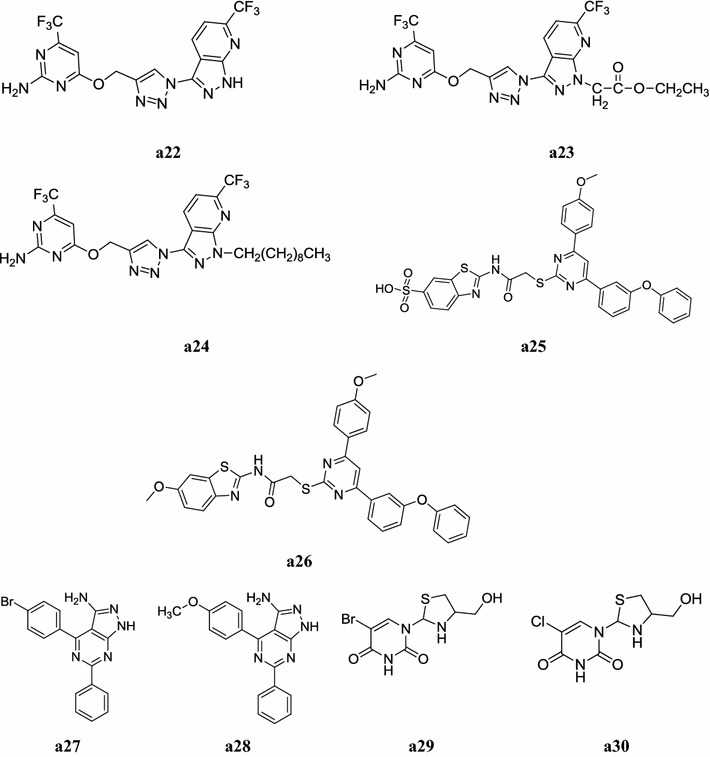
Chemical structure of the most active antimicrobial pyrimidine derivatives (**a22**–**a30**)

Nagender et al. developed a new series of novel pyrazolo[3,4-*b*]pyridine and pyrimidine functionalized 1,2,3-triazole derivatives using 6-trifluoro methylpyridine-2(1*H*) one and screened its antimicrobial activity against i.e. *Micrococcus luteus* MTCC 2470, *Staphylococcus aureus* MTCC 96, *Staphylococcus aureus* MLS-16 MTCC 2940, *Bacillus subtilis* MTCC 121, *Escherichia coli* MTCC 739, *Pseudomonas aeruginosa* MTCC 2453, *Klebsiella planticola* MTCC 530 and *Candida albicans* MTCC 3017. In this series, compounds, **a22**, **a23** and **a24** were displayed better antimicrobial activity but less than the standard drugs (ciprofloxacin) (Table 11, Fig. 4) [17].

**Table 11:** MIC values of the compounds **a22, a23** and **a24**

Compounds	Minimum inhibitory concentration (µg/ml)						
	*M. luteus*	*S. aureus*	*S. aureus*	*B. subtilis*	*E. coli*	*P. aeruginosa*	*K. planticola*
**a22**	7.8	15.6	15.6	15.6	7.8	7.8	15.6
**a23**	> 250	15.6	7.8	15.6	15.6	15.6	7.8
**a24**	15.6	7.8	7.8	15.6	7.8	7.8	7.8
Ciprofloxacin	0.9	0.9	0.9	0.9	0.9	0.9	0.9

Patel et al. synthesized a new series of pyrimidine derivatives and demonstrated its antimicrobial activity (Minimum inhibitory concentration) against four different strains, viz two Gram positive bacteria (*S. aureus* and *S. pyogenes*) and two Gram negative bacteria and (*E. coli* and *P. aeruginosa*) compared it with standard drugs ampicillin, chloramphenicol, ciprofloxacin and norfloxacin and antifungal activities against *C. albicans* and *A. niger* using nystatin as standard drug by broth dilution method, compounds, **a25** and **a26** were showed promising antimicrobial activity (Table 12, Fig. 4) [18].

**Table 12:** Antimicrobial activity of compounds **a25** and **a26**

Compounds	Microbial strains (µg/ml)					
	*E. coli*	*P. aeruginosa*	*S. aureus*	*S. pyogenus*	*C. albicans*	*A. niger*
**a25**	62.5	200	100	100	200	250
**a26**	25	50	100	50	500	250
Chloramphenicol	50	50	50	50	–	–
Ciprofloxacin	25	25	50	50	–	–
Norfloxacin	10	10	10	10	–	–
Nystatin					100	100

A new library of pyrazolo[3,4-*d*]pyrimidine derivatives was synthesized by Rostamizadeh et al. and screened for its antibacterial activity against two Gram-negative strains of bacteria: *Pseudomonas aeruginosa* and *Klebsiella pneumonia* and two Gram-positive bacteria: *Staphylococcus aureus* and *Enterococcus raffinosus* L. Amongst the tested compounds, compounds **a27** and **a28** exhibited higher antibacterial activity than the standard drugs (Table 13, Fig. 4) [19].

**Table 13:** Antibacterial activity of some novel pyrazolopyrimidine derivatives

Compounds	MIC (µmol/l)	
	*Enterococcus raffinosus*	*Staphylococcus aureus*
**a27**	12.3	3.8
**a28**	14.2	4.2
Penicillin G	93.5	24.4

Sriharsha et al. developed a new series of novel 1,3-thiazolidine pyrimidine derivatives and carried out its antibacterial activity against 14 bacterial strains i.e. *Citrobacter* sp., *Escherichia coli, Klebsiella* sp., *Proteus mirabilis, Pseudomonas aeruginosa, S. parathyphi A, S. parathyphi B, Salmonella typhi, S. typhimurium, Shigella boydii, Shigella flexneri, Shigella sonnei, Staphylococcus aureus* and *Streptococcus faecalis.* All compounds with free NH group in the pyrimidine moiety showed significant biological activity against all the standard strains used and in that compounds **a29** and **a30** showed promising activity against 14 human pathogens tested and compared with the ciprofloxacin and bacitracin used as standard drugs (Table 14, Fig. 4) [20].

**Table 14:** Antibacterial activity (zone of inhibition = mm) of most active compounds

S. no	Pathogens	**a29**	**a30**	Bacitracin	Ciprofloxacin
**1**	*Citrobacter* sp.	37.16 ± 0.15	28.66 ± 0.15	0.00 ± 0.00	19.62 ± 0.18
**2**	*Escherichia coli*	36.66 ± 0.15	27.83 ± 0.20	0.00 ± 0.00	0.00 ± 0.00
**3**	*Klebsiella* sp.	32.50 ± 0.13	25.50 ± 0.27	0.00 ± 0.00	20.25 ± 0.16
**4**	*Proteus mirabilis*	28.66 ± 0.25	23.33 ± 0.17	0.00 ± 0.00	18.25 ± 0.16
**5**	*Pseudomonas aeruginosa*	30.66 ± 0.12	27.83 ± 0.27	0.00 ± 0.00	34.25 ± 0.16
**6**	*S. parathyphi A*	34.66 ± 0.12	24.50 ± 0.12	0.00 ± 0.00	27.75 ± 0.16
**7**	*S. parathyphi B*	32.50 ± 0.13	27.83 ± 0.20	0.00 ± 0.00	27.63 ± 0.18
**8**	*Salmonella typhi*	29.50 ± 0.25	19.66 ± 0.11	0.00 ± 0.00	20.25 ± 0.16
**9**	*S. typhimurium*	34.66 ± 0.12	23.33 ± 0.17	0.00 ± 0.00	18.75 ± 0.31
**10**	*Shigella boydii*	37.50 ± 0.07	28.66 ± 0.25	0.00 ± 0.00	17.75 ± 0.16
**11**	*Shigella flexneri*	35.66 ± 0.08	25.50 ± 0.27	0.00 ± 0.00	27.63 ± 0.18
**12**	*Shigella sonnei*	32.50 ± 0.13	37.50 ± 0.07	0.00 ± 0.00	21.75 ± 0.16
**13**	*Staphylococcus aureus*	37.50 ± 0.07	32.50 ± 0.13	26.75 ± 0.84	18.13 ± 0.48
**14**	*Streptococcus faecalis*	38.50 ± 0.12	35.66 ± 0.08	0.00 ± 0.00	0.00 ± 0.00

### Anticancer activity

Cancer is a multifaceted disease that represents one of the leading causes of mortality in developed countries. Worldwide, one in eight deaths are due to cancer and it is the second most common cause of death in the US, exceeded only by heart disease. Chemotherapy is the mainstay for cancer treatment, the use of available chemotherapeutics is often limited due to undesirable side effects. It is important to identify new molecules and new targets for the treatment of cancer [17].

**Fig. 5:**
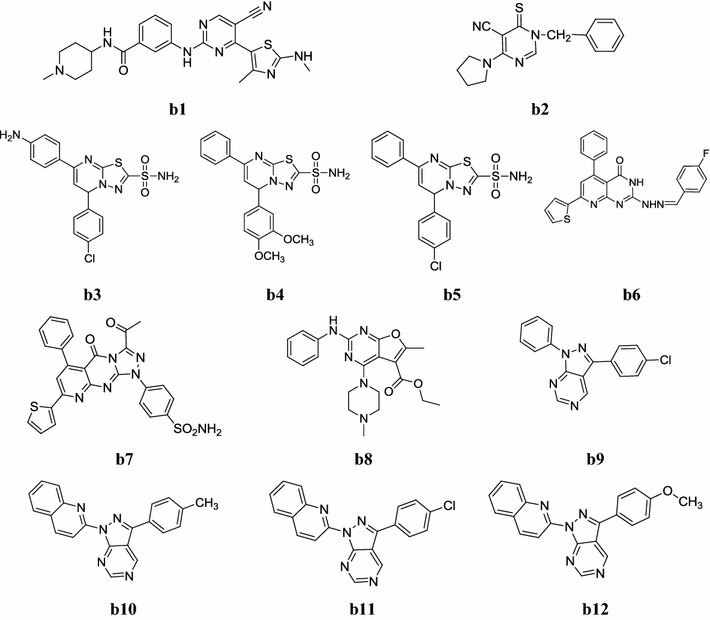
Chemical structures of the most active anticancer pyrimidine derivatives (**b1**–**b12**)

Shao et al. synthesized a new derivatives of 2,4,5-trisubstituted pyrimidine CDK inhibitors as potential antitumour agents. The synthesized 2,4,5-trisubstituted pyrimidine derivatives were evaluated for their antitumour activity against a panel of cancer cell lines including colorectal, breast, lung, ovarian, cervical and pancreatic cancer cells. Among the synthesized derivatives, compound **b1**, possessing appreciable selectivity for CDK9 over other CDKs, is capable of activating caspase 3, reducing the level of Mcl-1 anti-apoptotic protein and inducing cancer cell apoptosis (Table 15, Fig. 5) [21].

**Table 15:** Anti-proliferative activity of **b1** in human cancer cell lines

Compound	Human cancer cell lines		
	Origin	Designation	48 h-MTT GI50 (µM) ± SD
**b1**	Colon carcinoma	HCT-116	0.79 ± 0.08
	Breast carcinoma	MCF-7	0.64 ± 0.08
		MDA-MB468	1.51 ± 0.34
	Lung carcinoma	A549	2.01 ± 0.55
	Ovarian carcinoma	A2780	1.00 ± 0.11
	Cervical carcinoma	HeLa	0.90 ± 0.07
	Pancreatic carcinoma	Miacapa-2	1.25 ± 0.26

Cocco et al. synthesized a new class of 6-thioxopyrimidine derivatives and its molecular structures were confirmed by IR, NMR and elemental analyses study. The synthesized derivatives were evaluated their in vitro anticancer potential against multiple panels of 60 human cancer cell lines by Sulforhodamine B assay. All synthesized 6-thioxopyrimidine derivatives exhibited good anticancer potential, especially, compound **b2** showed the best cytotoxicity (Table 16, Fig. 5) [2].

**Table 16:** Anticancer activity results of most active compound **b2**

Compound	CNS cancer cell lines	10^−5^ M concentration	Ovarian cancer cell lines	10^−5^ M concentration
**b2**	SF-268	2.95	IGROV1	7.71
	SF-295	9.79	OVCAR-3	6.34
	SF-539	3.99	OVCAR-4	3.42
	SNB-19	5.42	OVCAR-8	4.92
	SNB-57	2.49	–	–
	U-251	3.58	–	–

A new library of sulfonamide derivatives was synthesized and investigated for its in vitro and in vivo antitumor potential by El-Sayed et al. Preliminary biological study revealed that compounds, **b3**, **b4** and **b5** showed the highest affinity to DNA and highest percentage increase in lifespan of mice inoculated with Ehrlich ascites cells over 5-flurouracil was taken as standard drug (Table 17, Fig. 5) [22].

**Table 17:** In vitro anticancer activity results of active compounds

Group	Normal	Control (Ehrlich only)	**b3**	**b4**	**b5**	5-Fluorouracil
% Increase in lifespan over control	71.43	0	71.43	57.14	42.86	42.86

Two new class of pyrido[2,3-*d*]pyrimidine and pyrido[2,3-*d*][1,2,4]triazolo[4,3-*a*] pyrimidines were synthesized by Fares et al. The molecular structures of synthesized derivatives were confirmed by physicochemical properties and spectral data (IR, NMR, Mass and elemental analyses) and screened for their anticancer activity against human cancer cell lines i.e. PC-3 prostate and A-549 lung. Some of the tested compounds exhibited high growth inhibitory potential against PC-3 cell, among them, compounds, **b6** and **b7** showed relatively potent antitumor potential (Table 18, Fig. 5) [23].

**Table 18:** Anticancer activity results of compounds **b6** and **b7**

Compounds	Cancer cell lines (IC_50_ = µM)	
	A-549	PC-3
**b6**	3.36 ± 0.39	1.54 ± 0.19
**b7**	0.41 ± 0.03	0.36 ± 0.02
5-Fluorouracil	4.21 ± 0.39	12.00 ± 1.15

Hu et al. developed a new library of 2,4-diamino-furo[2,3-*d*]pyrimidine and carried out its in vitro anticancer activity against A459 and SPC-A-1 cancer cell lines. Their structures were confirmed by 1H-NMR, EI-Ms, IR and elemental analysis. Among them, compound **b8**: ethyl-6-methyl-4-(4-methylpiperazin-1-yl)-2-(phenylamino)furo[2,3-*d*] pyrimidine-5-carboxylate was found to be most anticancer one against lung cancer cell line (A459 with IC_50_ 0.8 µM) (Fig. 5) [24].

Huang et al. developed a new series of pyrazolo[3,4-*d*]pyrimidines using 5-aminopyrazoles with formamide in presence of PBr_3_ as the coupling agent and its chemical structures were characterized by IR, ^1^H/^13^C-NMR, Mass, elemental analyses data. The synthesized compounds were screened their in vitro antiproliferative potential by MTT assay against human cancer cell line viz. NCI-H226 (lung carcinoma) and NPC-TW01 (nasopharyngeal carcinoma). From this series, compounds, **b9**, **b10**, **b11** and **b12** possessed better potency against NCI-H226 and NPC-TW01 cancer cells (Table 19, Fig. 5) [25].

**Table 19:** Antiproliferative results of active compounds (**b9**–**b12**)

Compounds	Cancer cell lines (GI_50_ = µM)	
	NCI-H226	NPC-TW01
**b9**	18	23
**b10**	29	30
**b11**	39	35
**b12**	37	36

**Fig. 6:**
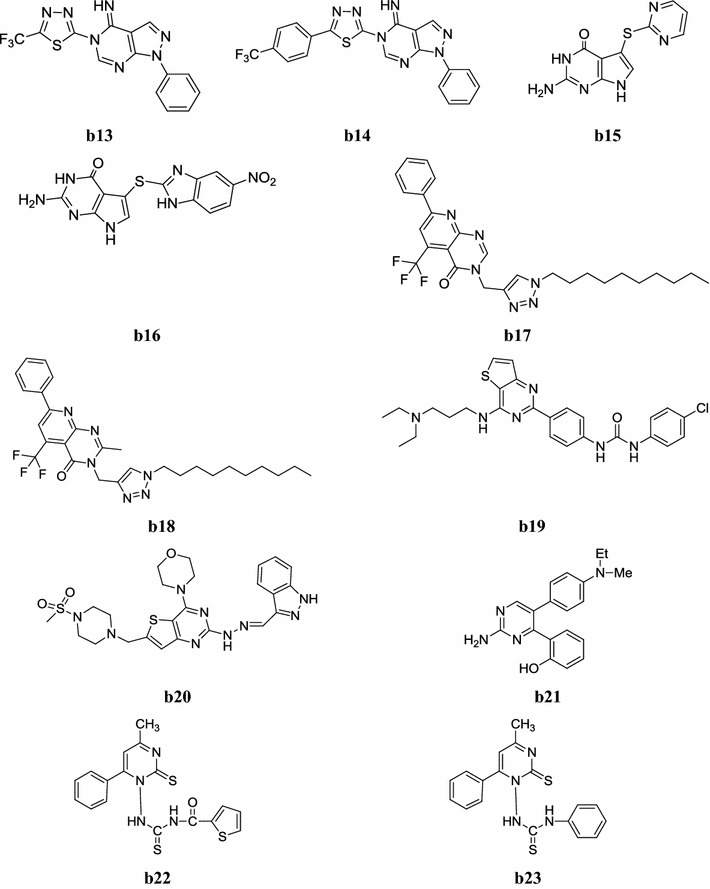
Chemical structures of the most active anticancer pyrimidine derivatives (**b13**–**b23**)

Song et al. synthesized a new library of fluorinated pyrazolo[3,4-*d*]pyrimidine derivatives by microwave (MW) irradiation method and evaluated its in vitro antitumor potential against human leukaemia (HL-60) cancer cell line by MTT assay. The preliminary results demonstrated that some of compounds exhibited potent antitumor inhibitory potential than doxorubicin (standard drug), especially compounds, **b13** and **b14** exhibited higher antitumor activity due to presence of CF group in its molecule structure (Table 20, Fig. 6) [26].

**Table 20:** Antitumor potential results of compounds **b13** and **b14**

Compounds	Human leukaemia (HL-60) cancer cell IC_50_ = µmol/l
**b13**	0.08
**b14**	0.21
Doxorubicin	0.55

Tangeda and Garlapati, developed new molecules of pyrrolo[2,3-*d*]pyrimidine and screened its in vitro anticancer activity against HCT116 colon cancer cell line. Especially, compounds, **b15** and **b16** were found to be most potent ones against HCT116 cell line with IC_50_ value of 17.61 and 17.60 µM respectively which is comparable with 5-fluorouracil (IC_50_ = 3.03 µM) (Fig. 6) [27].

Kurumurthy et al. prepared a novel class of alkyltriazole tagged pyrido[2,3-*d*] pyrimidine derivatives and its molecular structure were confirmed by IR, NMR, Mass and elemental analyses. The synthesized derivatives were evaluated their in vitro anticancer activity against three cancer cell lines i.e. U937 (human leukemic monocytic lymphoma), THP-1 (human acute monocytic leukemia) and Colo205 (human colorectal cancer) using MTT assay. Among the synthesized molecules, compounds **b17** and **b18** exhibited better anticancer activity than the standard etoposide (Table 21, Fig. 6) [28].

**Table 21:** In vitro cytotoxicity of pyrido[2,3-*d*]pyrimidine derivatives against U_937_, THP-1 and Colo205 cancer cell lines

Compounds	IC_50_ (µg/ml)		
	U_937_	THP-1	Colo205
**b17**	8.16 ± 0.68	16.91 ± 1.42	19.25 ± 1.46
**b18**	6.20 ± 0.68	11.27 ± 1.67	15.01 ± 1.54
Etoposide (positive control)	17.94 ± 1.19	2.16 ± 0.15	7.24 ± 1.26

Liu et al. synthesized two series of thieno[3,2-*d*]pyrimidine molecules containing diaryl urea moiety and screened their anticancer potential. The preliminary investigation showed that most compounds displayed good to excellent potency against four tested cancer cell lines compared with GDC-0941 and sorafenib as standard drugs. In particular, the most promising compound **b19** showed the most potent antitumor activities with IC_50_ values of 0.081, 0.058, 0.18 and 0.23 µM against H460, HT-29, MKN-45 and MDA-MB-231 cell lines, respectively (Fig. 6) [29].

Zhu et al. developed a series of 2,6-disubstituted-4-morpholinothieno[3,2-*d*]pyrimidine molecules and demonstrated its in vitro cytotoxic activity against H460, HT-29, MDA-MB-231, U87MG and H1975 cancer cell lines. Most of the target compounds exhibited moderate to excellent activity to the tested cell lines. The most promising compound **b20** is more active than the standard drug (Table 22, Fig. 6) [30].

**Table 22:** Cytotoxicity of compound **b20**

Compounds	IC_50_ = (µmol/l)				
	H460	HT29	MDA-MB-231	U87MG	H1975
**b20**	0.84	0.23	2.52	1.80	28.82
PAC-1	3.57	0.97	6.11	ND	ND

*ND* not determined

2,4,5-Substituted pyrimidine molecules were prepared and evaluated for their anticancer activity against different human cancer cell lines (A549, Calu-3, H460, SK-BR3, SGC-7901 and HT29) by Xie et al. Among the synthesized molecules, compounds **b21** showed good inhibition of several different human cancer cell lines with IC_50_ values from 0.024 to 0.55 µM (Table 23, Fig. 6) [31].

**Table 23:** In vitro anticancer activity of compound **b21**

Compound	Human cancer cell lines (IC_50_ = µM)					
	A549	Calu-3	H460	SK-BR3	SGC-7901	HT29
**b21**	0.55	0.50	0.12	0.30	0.30	0.090
Adriamycin	0.025	–	–	–	–	0.018
Docetaxel	–	0.10	0.0097	–	0.0084	–
GW572016	–	–	–	0.017	–	–

Al-Issa, developed a new series of fused pyrimidines and related heterocycles and evaluated its in vitro antitumor activity against human liver cancer cell line (HEPG2). Structures of all synthesized compounds were supported by spectral and elemental analyses. Among the synthesized compounds, compounds **b22** and **b23** showed significant in vitro antitumor activity (IC_50_, 17.4, 23.6 µg/ml) (Fig. 6) [32].

**Fig. 7:**
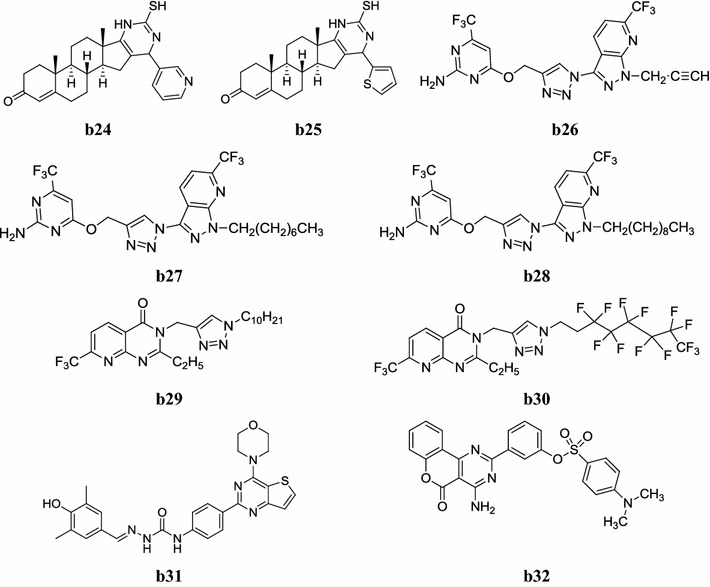
Chemical structures of the most active anticancer pyrimidine derivatives (**b24**–**b32**)

Mohareb et al. developed a new class of fused pyran, pyrimidine and thiazole molecules and evaluated its in vitro anticancer potential against cancer cell lines i.e. NUGC- gastric; DLDI-colon; HA22T-liver; HEPG2-liver; HONEI-nasopharyngeal carcinoma; HR-gastric; MCF-breast and WI38-normal fibroblast cells. In this study, compounds, **b24** and **b25** exhibited more anticancer potential (Table 24, Fig. 7) [33].

**Table 24:** Anticancer activity results of **b24** and **b25**

Compounds	Cytotoxicity (IC_50_ in nM)						
	NUGC	DLDI	HA22T	HEPG2	HONEI	MCF	WI38
**b24**	180	740	234	837	644	269	Na
**b25**	40	64	82	328	260	173	Na
CHS 828	25	2315	2067	1245	15	18	Na

A new series of novel pyrazolo[3,4-*b*]pyridine and pyrimidine functionalized 1,2,3-triazole derivatives were prepared from 6-trifluoro methyl pyridine-2(1*H*)one by Nagender et al. and screened for its cytotoxicity against four human cancer cell lines such as A549-Lung (CCL-185), MCF7-Breast (HTB-22), DU145-Prostate (HTB-81) and HeLa-Cervical (CCL-2). Among them, compounds, **b26**, **b27** and **b28** showed promising cytotoxicity (Table 25, Fig. 7) [17].

**Table 25:** In vitro cytotoxicity of most active compounds

Compounds	IC_50_ values (in µM)			
	A549	MCF7	DU145	HeLa
**b26**	4.1 ± 0.12	–	4.7 ± 0.18	–
**b27**	5.7 ± 0.22	24.7 ± 0.16	6.3 ± 0.21	22.7 ± 0.11
**b28**	4.2 ± 0.31	37.2 ± 0.31	5.8 ± 0.14	34.3 ± 0.32
5-Fluorouracil	1.3 ± 0.11	1.4 ± 0.09	1.5 ± 0.12	1.3 ± 0.14

Kumar et al. developed a new library of triazole/isoxazole functionalized 7-(trifluoromethyl)pyrido[2,3-*d*]pyrimidine derivatives and screened their anticancer activity against four human cancer cell lines using nocodazole as standard. Compounds **b29** and **b30** showed highest activity against PANC-1 (pancreatic cancer) and A549 (lung cancer) cell lines respectively (Table 26, Fig. 7) [34].

**Table 26:** Anticancer activity of triazole/isoxazole functionalized pyridopyrimidine derivatives

Compounds	GI_50_ values in µM			
	MDA MB-231	PANC1	A549	HeLa
**b29**	2.21 ± 0.08	0.02 ± 0.01	0.86 ± 0.03	0.81 ± 0.02
**b30**	2.83 ± 0.05	0.73 ± 0.01	0.03 ± 0.01	0.93 ± 0.03
Nocodazole	0.042 ± 0.001	0.029 ± 0.003	0.08 ± 0.001	0.063 ± 0.002

A new class of novel thieno[3,2-*d*]pyrimidine derivatives was synthesized by Liu et al. and studied for its anticancer potential against selected cancer cell lines viz: H460, HT-29, MKN-45 and MDA-MB-231. Most of compounds displayed good to excellent potency against four tested cancer cell lines as compared with GDC-0941 and sorafenib.

In this study, compound **b31** was found to be most active anticancer one (Table 27, Fig. 7) [35].

**Table 27:** Cytotoxicity of compound **b31**

Compound	IC_50_ (µmol/l) ± SD			
	H460	HT-29	MKN-45	MDA-MB-231
**b31**	0.057 ± 0.011	0.039 ± 0.008	0.25 ± 0.019	0.23 ± 0.020
GDC-0941	0.87 ± 0.20	0.86 ± 0.081	0.60 ± 0.12	0.28 ± 0.06
Sorafenib	2.19 ± 0.11	3.61 ± 0.36	2.32 ± 0.35	0.94 ± 0.13

Lv et al. synthesized a new series of 2-phenylpyrimidine coumarin derivatives and evaluated its in vitro antiproliferative activity against CNE2, KB and Cal27 cancer cell lines. The results showed that most of the derivatives had a favorable effect on resisting tumor cell proliferation, among them, compound **b32** exhibited the best antiproliferative activity and comparable to the standard drug (Table 28, Fig. 7) [36].

**Table 28:** In vitro anticancer activity of the synthesized compound

Compound	IC_50_ (µM)		
	CNE2	KB	Cal27
**b32**	1.92 ± 0.13	3.72 ± 0.54	1.97 ± 0.51
Doxorubicin	2.12 ± 0.56	3.04 ± 0.87	1.56 ± 0.64

### Antiviral activity

Antiviral nucleoside compounds inhibit viral genome replication by acting as mimetics of the natural nucleosides. Nucleoside analogues (NAs) can either act as chain terminators after being incorporated into growing DNA/RNA strands and/or inhibit the viral polymerase function by competition with the natural nucleoside 50-triphosphate substrate [3].

**Fig. 8:**
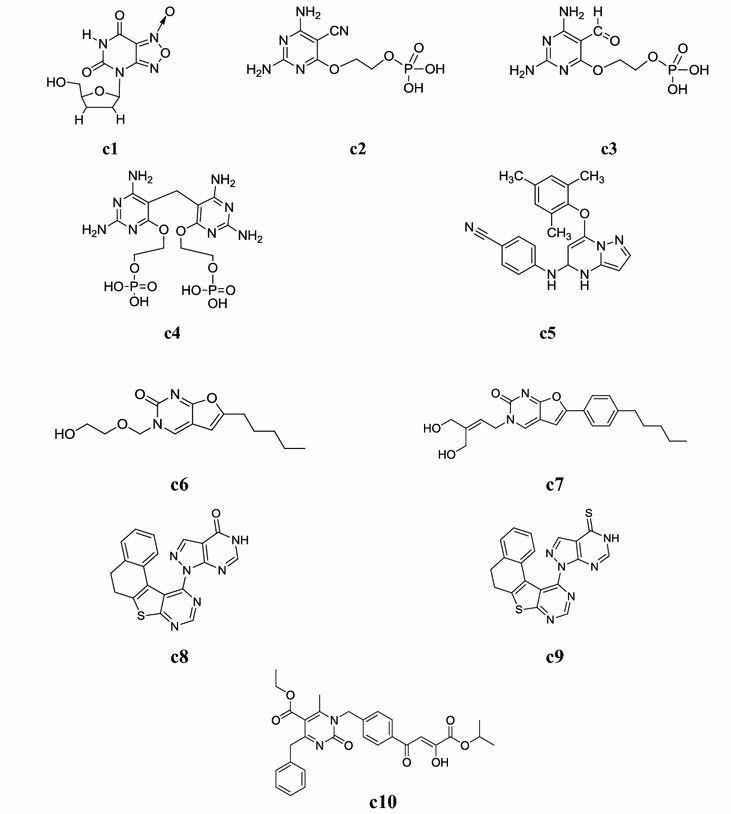
Chemical structures of the most active antiviral pyrimidine derivatives (**c1**–**c10**)

A new library of 4*H*,6*H*-[1,2,5]oxadiazolo[3,4-*d*]pyrimidine-5,7-dione 1-oxide nucleoside was synthesized by Xu et al. and screened for its in vitro anti-vesicular stomatitis virus (VSV) activity in Wish cell. All the synthesized derivatives showed obvious anti-VSV potential whereas, compound **c1** with ribofuranoside enhanced the anti-VSV potential by approximately 10–18 times compared to didanosine and acyclovir (standard drugs), respectively (Table 29, Fig. 8) [37].

**Table 29:** Experimental antiviral results of compound **c1**

Compound	Toxicity for wish cells and antivirus effect (TC0 µmol/l)	ED_50_	Model 1	Model 2
**c1**	2095	78	148	100
Acyclovir	3414	1411	–	–
Didanosine	2646	792	–	–

Hockova et al. synthesized a new series of 2,4-diamino-5-cyano-6[2-(phosphono methoxy)ethoxy]pyrimidine derivatives and evaluated its antiviral activity. The 5-cyano and 5-formyl derivatives (**c2**–**c4**) showed pronounced antiretroviral activity, comparable to that of the reference drugs adefovir and tenofovir (Table 30, Fig. 8) [38].

**Table 30:** Antiviral activity results of test compounds (**c2**–**c4)** in cell culture

Compounds	EC_50_^a^ (µmol/ml)			CC_50_^b^
	HIV (III_B_)	HIV-2 (ROD)	MSV	(µmol/ml) (CEM)
**c2**	0.011	0.0045	0.0095	≥ 0.3
**c3**	0.0045	0.0027	0.021	≥ 0.3
**c4**	0.080	0.050	–	≥ 0.2
Adefovir	0.0033	0.0066	0.0022	0.056
Tenofovir	0.0012	0.0014	0.0046	0.14

^a^50% effective concentration; ^b^ 50% cytostatic concentration

Tian et al. developed a novel library of 5,7-disubstituted pyrazolo[1,5-*a*]pyrimidine molecules and carried out its anti-HIV potential. From the series, compound **c5**: 4-(7-(mesityloxy)-4,5-dihydropyrazolo[1,5-*a*]pyrimidin-5-ylamino)benzonitrile was found to be the most active one (Fig. 8) with an EC_50_ = 0.07 µM against wild-type HIV-1 and very high selectivity index (SI, 3999) than the reference drugs (nevirapine and delavirdine) [39].

A new class of novel acyclic nucleosides in the 5-alkynyl and 6-alkylfuro[2,3-*d*] pyrimidines was synthesized by Amblard et al. and screened for its antiviral activity against human immunodeficiency virus (HIV), herpes simplex virus (HSV-1). Compounds, **c6** and **c7** exhibited moderate antiviral activity (Table 31, Fig. 8) [40].

**Table 31:** Antiviral activity results (µM) of compounds **c6** and **c7**

Compounds	Anti-HIV-1 activity in PBMCs		HSV-1 plaque reduction assay	
	EC_50_	EC_90_	EC_50_	EC_90_
**c6**	2.7	19.8	6.3	16.4
**c7**	4.9	13.07	4.8	46.2
AZTa	0.016	0.20	> 10	> 10
Acyclovira	> 100	> 100	0.11	0.69

A series of pyrazole and fused pyrazolo pyrimidines was synthesized by Rashad et al. and studied for their antiviral activity against hepatitis-A virus (HAV) and herpes simplex virus type-1 (HSV-1). The substituted pyrazole and fused pyrazolopyrimidine derivatives, **c8** and **c9** revealed higher anti-HSV-1 activity at concentration of 10 µg/10^5^ cells and antiviral results are compared with amantadine and acyclovir (Fig. 8) [41].

Sari et al. developed a new library of dihydropyrimidine α,γ-diketobutanoic acid molecules and screened its antiviral potential. Among the series, compound **c10** ((*Z*)-ethyl-4-benzyl-1-(4-(3-hydroxy-4-isopropoxy-4-oxobut-2-enoyl)benzyl)-6-methyl-2-oxo-1,2-dihydro pyrimidine-5-carboxylate) was found to be most active anti-HIV agent (Table 32, Fig. 8) [42].

**Table 32:** Antiviral activity results of compound **c10**

Compound	EC_50_ (µM)
**c10**	17.2
AZT	0.0074

### Antimalarial activity

Malaria is the most serious and widespread parasitic disease because of its prevalence, virulence and drug resistance, having an overwhelming impact on public health in developing regions of the world. *Plasmodium falciparum* is the main cause of severe clinical malaria and death. Endemic mapping indicates that *P. falciparum* and *P. vivax* account for 95% of the malarial infections [43]. According to a WHO report, malaria accounted for 207 million cases and an estimated 627,000 deaths worldwide in 2013 [8].

**Fig. 9:**
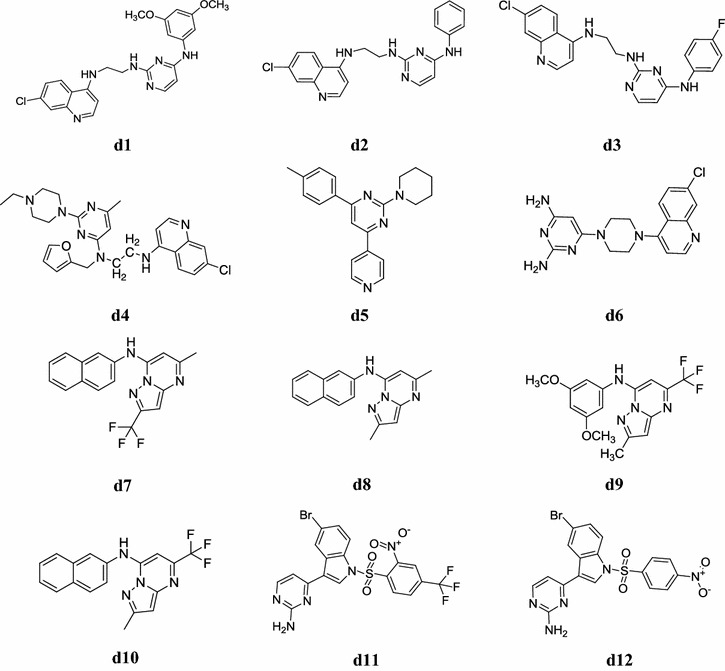
Chemical structures of the most active antimalarial pyrimidine derivatives (**d1**–**d12**)

Kumar et al. synthesized a new series of 4-aminoquinoline-pyrimidine hybrids and evaluated its antimalarial potential. Several compounds showed promising in vitro antimalarial activity against both CQ sensitive and CQ-resistant strains with high selectivity index. The in vitro evaluation of these hybrids against D6 and W2 strains of *P. falciparum* depicted the antimalarial activity in the nanomolar range. Also, these hybrids exhibited high selectivity indices and low toxicity against the tested cell lines. Compounds (**d1**, **d2** and **d3)** (Fig. 9) exhibited very potent antimalarial activity with IC_50_ = 0.033, 0.019 and 0.028 µM respectively which were comparable to the standard drug chloroquine (IC_50_ = 0.035 µM) against CQ-sensitive strain [8].

Maurya et al. developed a new series of novel *N*-substituted 4-aminoquinoline-pyrimidine hybrids via simple and economic route and evaluated its antimalarial activity. Most compounds showed potent antimalarial activity against both CQ-sensitive and CQ-resistant strains with high selectivity index. All the compounds were found to be non-toxic to the mammalian cell lines. The most active compound **d4** was analyzed for heme binding activity using UV spectrophotometer. Compound **d4** was found to interact with heme and a complex formation between compound **d4** and heme in a 1:1 stoichiometry ratio was determined using job plots. The interaction of these hybrids was also investigated by the molecular docking studies in the binding site of wild type Pf-DHFR-TS and quadruple mutant Pf-DHFR-TS (Table 33, Fig. 9) [44].

**Table 33:** In vitro antimalarial activity of AQ-furfural-2-carbaldehyde-pyrimidine hybrids

Compound	*P. falciparum* D6		*P. falciparum* W2		VERO cells	Resistance index
	IC_50_ (µM)	(SI)	IC_50_ (µM)	(SI)		
**d4**	0.038 ± 0.000	> 263.15	0.040 ± 0.001	> 250.0	NC	1.05
Chloroquine	0.011 ± 0.004	> 909.09	0.317 ± 0.051	> 31.54	NC	28.81
Pyrimethamine	0.009 ± 0.003	> 1111.1	NA	–	NC	–
Artemisinin	0.045 ± 0.001	> 222.22	0.023 ± 0.001	434.78	NC	0.511

Agarwal et al. developed a new series of 2,4,6-trisubstituted-pyrimidines and evaluated its in vitro antimalarial activity against *Plasmodium falciparum*. All the synthesized compounds showed good antimalarial activity against *Plasmodium falciparum* whereas, compound **d5** exhibited higher antimalarial activity than pyrimethamine used as standard drug (Table 34, Fig. 9) [43].

**Table 34:** Antimalarial in vitro activity against *P. falciparum*

Compound	MIC (µg/ml)
**d5**	0.25
Pyrimethamine	10

Pretorius et al. synthesized a new library of quinoline–pyrimidine hybrids and evaluated its in vitro antimalarial activity against the D10 and Dd2 strains of *Plasmodium falciparum*. The compounds were all active against both strains. However, hybrid (**d6**, Fig. 9) featuring piperazine linker stood as the most active of all. It was found as potent as CQ and PM against the D10 strain and possessed a moderately superior potency over CQ against the Dd2 strain (IC_50_: 0.157 vs 0.417 µM) and also displayed activity comparable to that of the equimolar fixed combination of CQ and PM against both strains [45].

Azeredo et al. synthesized a new series of 7-aryl aminopyrazolo[1,5-*a*]pyrimidine derivatives with different combinations of substituent’s at positions 2-,5- and 7- of the pyrazolo[1,5-*a*]pyrimidine ring. The compounds were tested against *Plasmodium falciparum*, as antimalarials in mice with *P. berghei* and as inhibitors of *Pf*DHODH. From this series, compounds, **d7**, **d8**, **d9** and **d10** were found to be the most active ones (Table 35, Fig. 9) [46].

**Table 35:** In vitro antimalarial activity results of active compounds

Compounds	(%) Activity *Pf*DHODH	IC_50_ against *Pf*DHODH (µM)
**d7**	67.474 ± 0.002	6 ± 1
**d8**	41 ± 3	4 ± 1
**d9**	77 ± 1	–
**d10**	60 ± 3	0.16 ± 0.01

A series of *N*-aryl and heteroaryl sulfonamide derivatives of meridianins were prepared by Yadav et al. and screened for its antimalarial activity against D6 and W2 strains of *Plasmodium falciparum*. Especially, compounds, **d11** and **d12** displayed promising antiplasmodial activity and comparable to the standard drugs (Table 36, Fig. 9) [47].

**Table 36:** In vitro antimalarial activity of *N*-aryl and heteroaryl sulfonamide derivatives

Compounds	*P. falciparum* (IC_50_ in µM (µg/ml))			
	*P. falciparum* (D6)		*P. falciparum* (W2)	
	IC_50_	SI	IC_50_	SI
**d11**	4.86 (2.3)	> 10.8	6.39 (3.02)	> 8.2
**d12**	2.56 (1.38)	> 18	3.41 (1.84)	> 13.5
Artemisinin	< 0.09 (< 0.03)	–	< 0.09 (< 0.03)	–
Chloroquine	< 0.08 (< 0.03)	–	0.72 (0.23)	–

### Anti-inflammatory activity

Non-steroidal anti-inflammatory drugs (NSAIDs) are among the most widely used therapeutics, primarily for the treatment of pain, rheumatic arthritis and various types of inflammatory conditions. However, their use is mainly restricted by their well known and serious adverse gastrointestinal side effects such as gastroduodenal erosions, ulcerations and nephrotoxicity [6].

**Fig. 10:**
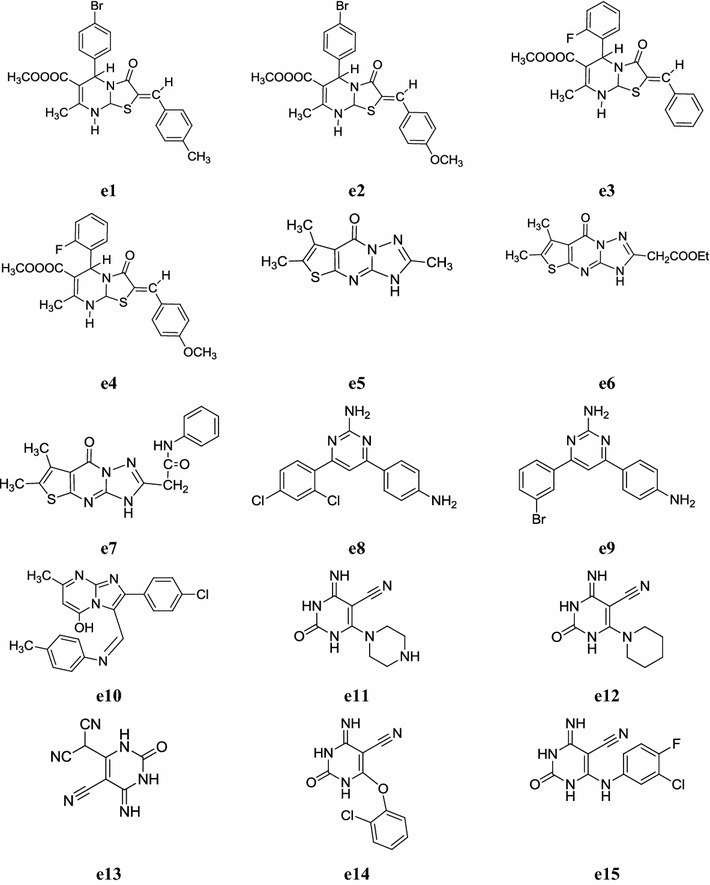
Chemical structures of the most active anti-inflammatory pyrimidine derivatives (**e1**–**e15**)

Tozkoparan et al. synthesized a new class of 2-benzylidene-7-methyl-3-oxo-5-(substituted phenyl)-2,3-dihydro-5*H*-thiazolo[3,2-*a*]pyrimidine-6-carboxylic acid methyl esters and evaluated its anti-inflammatory activity by carrageenan induced edema test using indomethacin as reference drug. Test results revealed that compounds, **e1**, **e2**, **e3**, **e4** exerted moderate anti-inflammatory activity at the 100 mg/kg dose level compared with indomethacin (Table 37, Fig. 10) [5].

**Table 37:** Anti-inflammatory activity in percentage (%) of synthesized compounds (**e1**–**e4**)

Compounds	Anti-inflammatory activity (%)^a^
**e1**	41
**e2**	38
**e3**	16
**e4**	28
Indomethacin	32

^a^ 100 mg/kg p.o. (*n *= 6)

Two new series of thieno[2′,3′:4,5]pyrimido[1,2-*b*][1,2,4]triazines and thieno[2,3-*d*][1,2,4]triazolo[1,5-*a*]pyrimidines were synthesized by Ashour et al. and evaluated for their anti-inflammatory and analgesic activity using diclofenac as reference drug. In general, the thieno[2,3-*d*][1,2,4]triazolo[1,5-*a*]pyrimidine derivatives exhibited better anti-inflammatory activity than the thieno[2′,3′5′:4,5]pyrimido[1,2-*b*][1,2,4]triazines. The thienotriazolo pyrimidine derivatives, **e5, e6** and **e7** (Fig. 10) were proved to display distinctive anti-inflammatory activity at the acute and sub acute models as well as good analgesic profile with a delayed onset of action. The anti-inflammatory screening results are presented in Tables 38 and 39 [6].

**Table 38:** Anti-inflammatory activity of compounds (**e5**–**e7**) in formal in induced rat paw edema bioassay (sub-acute inflammatory model)

Compounds	Volume of edema (ml)^a^		
	0	1st day	8th day
**e5**	0.31 ± 0.01	0.51 ± 0.03^b^ (44)^c^	0.68 ± 0.02^b^ (31)
**e6**	0.35 ± 0.02	0.54 ± 0.01^b^ (47)	0.67 ± 0.02^b^ (40)
**e7**	0.33 ± 0.02	0.15 ± 0.01^b^ (50)	0.67 ± 0.02^b^ (37)
Control	0.32 ± 0.01	0.68 ± 0.01	0.86 ± 0.03
Diclofenac	0.32 ± 0.02	0.52 ± 0.02^b^ (44)	0.64 ± 0.02^b^ (40)

^a^Values are expressed as mean ± S.E. (Number of animals N = 5 rats)

^b^Significantly different compared to corresponding control P ≤ 0.05

^c^Between parentheses (percentage anti-inflammatory activity %)

**Table 39:** Anti-inflammatory activity of the fused thienopyrimidines in formalin-induced rat paw edema bioassay (acute inflammatory model)

Compounds	Volume of edema (ml)^a^				ED_50_ (mg/kg)
	0	1 h	2 h	4 h	
**e5**	0.31 ± 0.01	0.44 ± 0.02^b^ (38)^c^	0.49 ± 0.01^b^ (43)	0.52 ± 0.02^b^ (52)	23.45^d^
**e6**	0.35 ± 0.02	0.46 ± 0.01^b^ (47)	0.50 ± 0.01^b^ (53)	0.54 ± 0.02^b^ (56)	28.15
**e7**	0.33 ± 0.02	0.46 ± 0.01^b^ (42)	0.53 ± 0.01^b^ (37)	0.59 ± 0.02^b^ (40)	26.12
Control	0.32 ± 0.01	0.55 ± 0.01	0.64 ± 0.02	0.76 ± 0.01	–
Diclofenac	0.32 ± 0.02	0.45 ± 0.01^b^ (38)	0.50 ± 0.02^b^ (43)	0.53 ± 0.02^b^ (52)	25.13

^a^Values are expressed as mean ± SE (number of animals N = 5 rats)

^b^Significantly different compared to corresponding control P ≤ 0.05

^c^Between parentheses (percentage anti-inflammatory activity %)

^d^ED_50_ is the effective dose calculated after 2 h

Yejella and Atla, synthesized a new series of 2,4,6-trisubstituted pyrimidines and screened its in vivo anti-inflammatory activity by carrageenan induced rat paw edema model. Compounds, **e8**: 2-amino-4-(4-aminophenyl)-6-(2,4-dichlorophenyl)pyrimidine and **e9**: 2-amino-4-(4-aminophenyl)-6-(3-bromophenyl)pyrimidine were found to be the most potent anti-inflammatory agents compared with ibuprofen (Table 40, Fig. 10) [48].

**Table 40:** Anti-inflammatory activity of pyrimidine derivatives

Comp.	Percent inhibition ± SEM at various time intervals					
	0.5 h	1.0 h	2.0 h	3.0 h	4.0 h	6.0 h
**e8**	15.22 ± 0.68*	50.45 ± 1.23*	87.23 ± 2.61*	62.51 ± 2.33*	56.94 ± 1.79	48.39 ± 2.65
**e9**	18.26 ± 0.68*	49.35 ± 1.41*	86.99 ± 2.62*	62.13 ± 2.25*	53.32 ± 2.01	42.11 ± 2.75
Ibuprofen	20.26 ± 0.90*	53.95 ± 0.97*	97.09 ± 2.86*	79.97 ± 2.38*	67.93 ± 2.22*	58.02 ± 1.87*

All values are represented as mean ± SEM (*n *= 6). *P < 0.01 compared to saline control group. One-way ANOVA, Dunnett’s *t* test. Dosage: Ibuprofen-10 mg/kg and test compounds-10 mg/kg body weight by orally

Zhou et al. synthesized a new series of imidazo[1,2-*a*]pyrimidine derivatives and screened its anti-inflammatory potential with selective cyclooxygenase-2 (COX-2) inhibitors. In this series, compound **e10** exhibited potent activity (63.8%) than ibuprofen (44.3%). The human whole blood assay still revealed that **e10** (Fig. 10) has selective COX-2 inhibition (IC_50_ = 13 µmol/l) which is 13 times more potent than its inhibitory activity to COX-1 (IC_50_ = 170 µmol/l) and swollen inhibition 63.8%. The results indicated that imidazo[1,2-*a*] pyrimidine compounds keep moderate anti-inflammatory activity as compared to ibuprofen (standard drug) [49].

Gondkar et al. prepared a new class of substituted 1,2,3,4-tetrahydropyrimidine and screened its in vitro anti-inflammatory activity by inhibition of protein denaturation method using diclofenac (standard drug). The results revealed that almost all the tested compounds showed potent anti-inflammatory potential. All synthesized derivatives were tested their in vitro anti-inflammatory activity using inhibition of albumin denaturation technique compared to standard diclofenac. Derivatives, **e11**, **e12**, **e13**, **e14** and **e15** (Fig. 10) showed significant in vitro anti-inflammatory activity with % inhibition of albumin denaturation 98, 97, 90, 94, and 96% respectively [50].

**Fig. 11:**
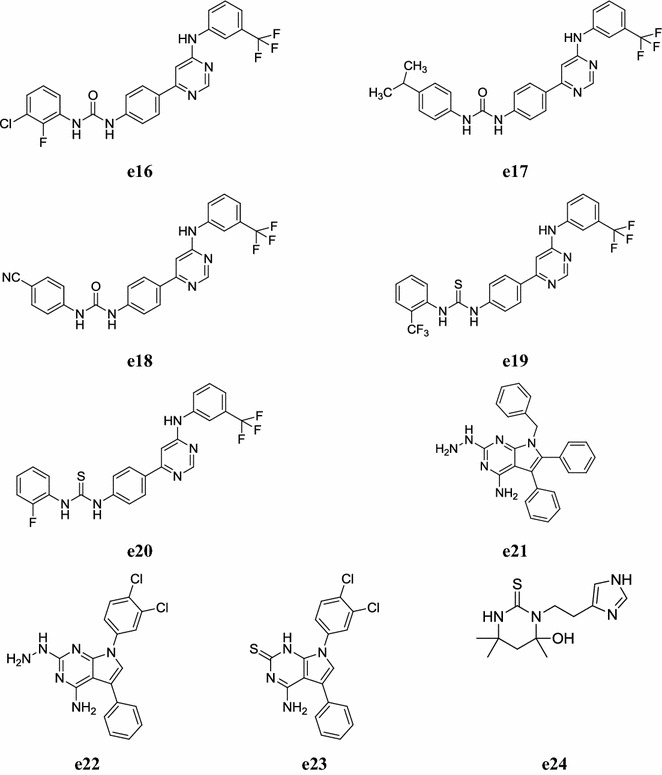
Chemical structures of the most active anti-inflammatory pyrimidine derivatives (**e16**–**e24**)

Keche et al. developed a new series of novel 4-(3-(trifluoromethyl)phenylamino-6-(4-(3-arylureiodo/arylthioureido/arylsulfonamido)-pyrimidine derivatives by the sequential Suzuki cross coupling and screened for their anti-inflammatory activity. Among all the synthesized derivatives, compounds, **e16**, **e17**, **e18**, **e19**, **e20** and **e21** were found to have moderate to potent anti-inflammatory activity and compared to dexamethasone used as reference drug (Table 41, Fig. 11) [51].

**Table 41:** Anti-inflammatory activity of novel pyrimidine derivatives

Compounds	% Inhibition at 10 µM NF-α	IL-6
**e16**	78	96
**e17**	71	90
**e18**	61	80
**e19**	68	82
**e20**	50	62
Dexamethasone	72	86

Mohamed et al. synthesized a new library of thio containing pyrrolo[2,3-*d*]pyrimidine derivatives and carried out its in vitro anti-inflammatory potential using the carrageenan-induced rat paw oedema assay. The potency and duration of action was compared to ibuprofen was taken as standard drug. From tested compounds, compounds **e21**, **e22** and **e23** showed best anti-inflammatory activity (Table 42, Fig. 11) [52].

**Table 42:** In vivo anti-inflammatory activity

Compounds	Oedema induced by carrageenan (% oedema % inhibition relative to control)							
	1 h		2 h		3 h		4 h	
	Swel	% inh	Swel	% inh	Swel	% inh	Swel	% inh
**e21**	0.206	10.43	0.101	61.15	0.142^c^	73.9	0.132^b^	79.04
**e22**	0.196	14.78	0.182	30	0.022^c^	95.58	0.282	67.43
**e23**	0.216	6.08	0.012^b^	95.38	0.024^c^	95.95	0.202^a^	76.82
Ibuprofen	0.216	6.08	0.14	45	0.214^b^	60.66	0.192^a^	69.52

As indicated: ^a^ P < 0.05; ^b^ P < 0.01; ^c^ P < 0.001

Sondhi et al. synthesized new derivatives of pyrimidine and screened their anti inflammatory activity carried out using carrageenin-induced paw oedema assay. All compounds exhibited good activity whereas, compound **e24** was found to be most active one comparable to the standard drug ibuprofen (Table 43, Fig. 11) [53].

**Table 43:** Anti-inflammatory of compound **e24**

Compound	Dose mg/kg po	Anti-inflammatory activity %
**e24**	100	65
Ibuprofen	100	66.8

### Antioxidant activity

Oxidative stress seems to play a significant role in various human diseases, including cancers. Antioxidant compounds are the agents that neutralize free radicals, which scavenge reactive oxygen species, may have potent value in preventing the onset and propagation of oxidative diseases such as neurovascular, cardiovascular diseases. Pyrimidine and its derivatives have recently attracted the attention of medicinal chemists in exploring their potential as antioxidant agents [1].

**Fig. 12:**
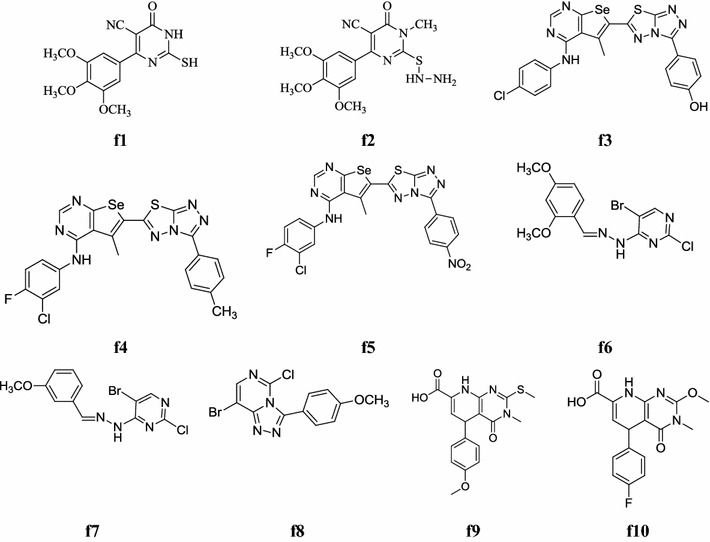
Chemical structures of the most active antioxidant pyrimidine derivatives (**f1**–**f10**)

Bhalgat et al. developed a new class of novel pyrimidines and its triazole fused derivatives and investigated its in vitro antioxidant by various methods as scavenging of hydrogen peroxide, scavenging of nitric oxide radical and lipid per oxidation inhibitory activity. Compounds, **f1** showed good antioxidant activity as compared to standard by scavenging of nitric oxide radical and hydrogen peroxide, while **f2** showed most potent antioxidant activity by scavenging of nitric oxide (Table 44, Fig. 12) [7].

**Table 44:** Antioxidant activity (IC-50 values) of compounds **f1** and **f2**

Compound	IC-50 (mean ± SD)^a^ (µg/ml)		
	Scavenging of nitric oxide radical	Scavenging of hydrogen peroxide	Lipid peroxidation inhibitory activity
**f1**	51 ± 0.058	41 ± 0.087	40 ± 0.121
**f2**	47 ± 0.052	52 ± 0.279	43 ± 0.333
Standard	56 ± 0.087	38 ± 0.121	26 ± 0.333

^a^Average of three determination

Kotaiah et al. synthesized new molecules of novel 1,2,4-triazolo[3,4-*b*][1,3,4]thiadiazol-6-yl)selenopheno[2,3-*d*]pyrimidines with substituted anilines and benzoic acid. The antioxidant activity of the synthesized compounds was evaluated by DPPH, NO and H_2_O_2_ radical scavenging methods. In this series, compounds, **f3**, **f4** and **f5** showed promising antioxidant activity compared to standard drug (Table 45, Fig. 12) [54].

**Table 45:** Antioxidant activity of most compounds

Compounds	Scavenging activity (IC_50_ µg/ml)		
	DPPH	NO	H_2_O_2_
**f3**	11.02 ± 0.27	13.72 ± 1.26	15.38 ± 0.96
**f4**	10.41 ± 0.23	12.74 ± 0.18	17.08 ± 0.12
**f5**	9.46 ± 0.91	8.20 ± 1.60	12.54 ± 1.17
AA	12.27 ± 0.86	14.62 ± 0.97	15.24 ± 0.44
BHT	16.53 ± 1.74	19.06 ± 1.04	17.82 ± 0.28

Lower IC_50_ values indicate higher radical scavenging activity

*AA* ascorbic acid, *BHT* butylated hydroxy toluene

Mohana et al. reported a new series of pyrimidine derivatives and evaluated its antioxidant activity by DPPH method. The structures of all the new compounds are established on the basis of FT-IR, ^1^H-NMR and Mass spectral data. All the compounds showed DPPH radical scavenging activity, whereas, compounds, **f6**, **f7** and **f8** exhibited best radical scavengers due to presence of electron donating methoxy group at different position (*ortho, meta* and *para*) (Table 46, Fig. 12) [55].

**Table 46:** DPPH radical scavenging activity of the tested compounds

Compounds	Scavenging effect (%)		
	Concentration of the tested compounds (µg/ml)		
	100	150	200
**f6**	51.1	60.8	68.1
**f7**	35.2	46.3	52.1
**f8**	32.2	43.4	54.8
Ascorbic acid	73.0	85.3	98.2

Quiroga et al. developed a new library of 5-aryl-4-oxo-3,4,5,8-tetrahydropyrido[2,3-*d*] pyrimidine-7-carboxylic acids and carried out their antioxidant activity by DPPH (1,1-diphenyl-2-picryl-hydrazyl) radical scavenging assay. Compounds **f9** and **f10** showed antioxidant properties and compared to standard drugs (Table 47, Fig. 12) [56].

**Table 47:** Free radical scavenging (FRS_50_) for the tested pyrido[2,3-*d*]pyrimidines (**f9** and **f10**)

Compounds	FRS_50_ (µg/ml)	
	Mean	%RSD
**f9**	367	10
**f10**	472	10
Asc. acid	1.1	12
Quercetin	3.4	7

### Antileishmanial activity

Leishmaniasis, a vector-borne parasitic disease, is a major cause of concern in developing countries. The disease is caused by more than 20 species of protozoan *Leishmania* and transmitted by the bite of female phlebotomine sand flies. Leishmaniasis has traditionally been classified into three major clinical forms: visceral leishmaniasis (VL), cutaneous leishmaniasis (CL) and mucocutaneous leishmaniasis (MCL) which differs in immunopathologies and degree of morbidity and mortality. VL caused by *Leishmania donovani* is the most severe form of leishmaniasis and is usually fatal in the absence of treatment. Most of the first line drugs available for the treatment of leishmaniasis such as sodium stibogluconate, meglumine antimoniate, pentamidine etc. cause serious side effects and toxicity [57].

**Fig. 13:**
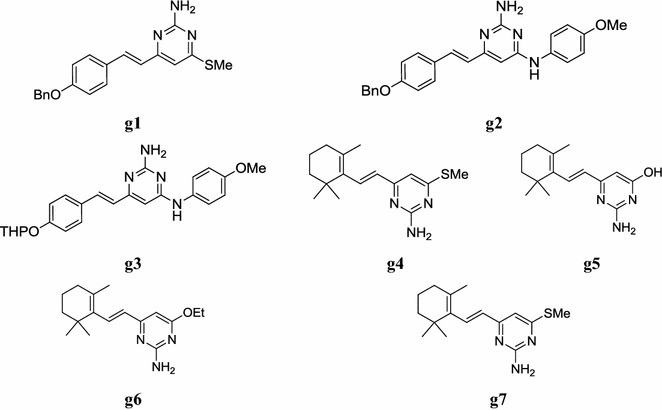
Chemical structures of the most active antileishmanial pyrimidine derivatives (**g1**–**g7**)

A new series of substituted aryl pyrimidine derivatives was synthesized by Suryawanshi et al. and evaluated for its in vitro antileishmanial potential against intracellular amastigotes of *Leishmania donovani* using reporter gene luciferase assay. All synthesized compounds showed promising IC_50_ values ranging from 0.5 to 12.9 µM. Selectivity indices (S.I.) of all these compounds are far better than sodium stibogluconate (SSG) and miltefosine used as standard drugs. On the basis of good selectivity indices compounds were further screened their in vivo antileishmanial activity against *L. donovani*/hamster model. Compounds, **g1**, **g2** and **g3** showed good inhibition (Table 48, Fig. 13) of parasitic multiplication that is 88.4, 78.1 and 78.2%, respectively at a daily dose of 50 mg/kg × 5 days, when administered intraperitoneally [57].

**Table 48:** In vitro and in vivo antileishmanial activity and cytotoxicity results of synthetic pyrimidine derivatives

Compounds	In vitro assessment		Selectivity index CC_50_/IC_50_	In vivo activity (dose—50 mg/kg × 5 days, ip^b^) % Inhibition ± SD
	IC_50_ (µM)	CC_50_ (µM)		
**g1**	2.0 ± 0.1	375.9 ± 5.1	188	88.4 ± 10.6
**g2**	0.5 ± 0.1	57.8 ± 5.9	116	78.1 ± 17.7
**g3**	2.7 ± 0.5	345.4 ± 19.6	128	78.2 ± 4.4
SSG^a^	59.8 ± 7.5	> 400 ± 0	> 7	88.5 ± 4.4
Miltefosine^c^	12.5 ± 0.9	54.7 ± 6.9	4	98.1 ± 1.0

IC_50_ and CC_50_ values are the mean ± SD of two independent experiments

The selectivity index is defined as the ratio of CC_50_ on vero cells to IC_50_ on *L. donovani* intramacrophagic amastigotes

^a^SSG = sodium stibogluconate (40 mg/kg × 5 days, ip)

^b^ip = intraperitonial; ^c^ Miltefosine (30 mg/kg × 5 days, po) used as a reference drugs

Pandey et al. synthesized some novel terpenyl pyrimidine from *α/β*-ionone keteneacetals and screened their in vivo leishmanicidal activity against amastigote stage of *Leishmania donovani* was determined in Golden hamsters (*Mesocricotus aurctus*) infected with HOM/IN/80/DD8 strain of *L. donovani*. The compounds, **g4**, **g5**, **g6** and **g7** showed promising in vivo antileishmanial activity (Table 49, Fig. 13) [58].

**Table 49:** Antileishmanial activity of compounds against amastigotes of *Leishmania donovani* in hamsters

Compounds	Dose (mg/kg)	In vivo inhibition (%)	
		Day-7	Day-28
**g4**	50	66	–
**g5**	50	22	63
**g6**	50	64	–
**g7**	50	64	–

### Miscellaneous activities

**Fig. 14:**
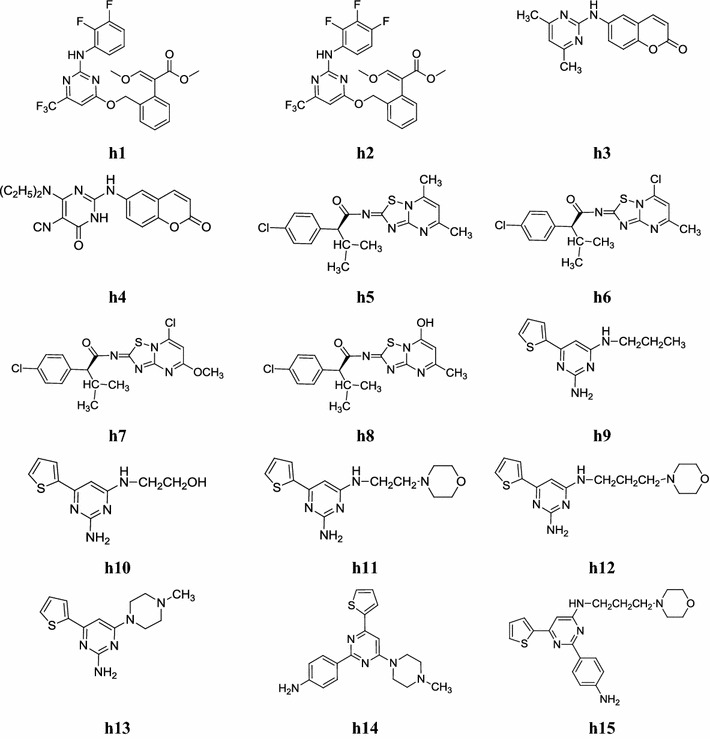
Chemical structures of the most active pyrimidine derivatives (**h1**–**h15**)

A new series of strobilurin-pyrimidine derivatives was synthesized by Chai et al. The synthesized compounds were evaluated for their acaricidal activity. Preliminary bioassays demonstrated that compounds, **h1** and **h2** exhibited significant control against *Tetranychus cinnabarinus* (Boisd.) at 0.625 mg/l, and their acaricidal potencies were higher than pyriminostrobin in a green house. Compounds, **h1** and **h2** (Fig. 14) were chosen as candidates for extensive greenhouse bioassays on larvae and eggs of *T. cinnabarinus.* Both of them showed potency consistent with pyriminostrobin against larvae and weaker potency than pyriminostrobin against eggs, as shown in Table 50 [59].

**Table 50:** Acaricidal activity of **h1** and **h2** against *T. cinnabarinus*

Compounds	*T. cinnabarinus*	(% mortality at given concentration mg/l)		
		10	25	0.625
**h1**	Larvae	100	98	77
	Eggs	100	70	25
**h2**	Larvae	100	100	100
	Eggs	75	20	10
Pyriminostrobin	Larvae	100	100	96
	Eggs	100	100	20

Amin et al. synthesized a new series of novel coumarin–pyrimidine hybrids and evaluated its vasorelaxant activity against nor-adrenaline-induced spasm on thoracic rat aorta rings and compared to prazocin (reference drug). From the series, compounds, **h3**: (6-(4,6-dimethylpyrimidin-2-ylamino)-2*H*-chromen-2-one) and **h4**: (6-(diethylamino)-5-isocyano-2-(2-oxo-2*H*-chromen-6-ylamino)pyrimidin-4(3*H*)-one) were found to be most prospective vasorelaxant agent with IC_50_ = 0.411 and IC_50_ = 0.421 mM respectively when compared with reference drug prazocin (IC_50_ = 0.487 mM). The chemical structure depicted in Fig. 14 [60].

Duan et al. designd and synthesized a new series of S(−)-2-(4-chlorophenyl)-*N*-(5,7-di- substituted-2*H*-[1,2,4]-thiadiazolo[2,3-*a*]pyrimidin-2-ylidene)-3-methylbutanamide derivatives. The synthesized compounds were evaluated for their herbicidal activity against three monocotyledon weeds and two dicotyledon weeds i.e. *Echinochloa crusgallis* L., *Sorghum bicolort*, *Digitaria sanguinalis* (L.) *scop Chenopodium serotinum* (L.) and *Amaranthus retroflexus* L., respectively. Compounds **h5** and **h6** showed the highest inhibitory activity against root and stalk of *Amaranthus retroflexus* L. in higher concentration (1.0 × 10^−4^ µg/ml), while compounds **h7** and **h8** showed good activity against root of *Echinochloa crusgallis* L. and stalk of *Chenopodium serotinum* L., respectively (Table 51, Fig. 14). The chiral target compounds showed improved herbicidal activity to some extent over their racemic counterparts against a variety of tested weeds, which might be contributed by the introduction of chiral active unit [61].

**Table 51:** The inhibition percentage of the target compounds against various weeds

Compounds	Concentration (ppm)	*Echinochloa crusgallis* L.		*Chenopodium serotinum* L.		*Amaranthus retroflexus* L.	
		Stalk	Root	Stalk	Root	Stalk	Root
**h5**	50	50	30	50	50	50	50
	100	80	30	80	85	95	100
**h6**	50	10	70	80	80	90	85
	100	30	90	85	90	100	100
**h7**	50	60	80	10	10	0	0
	100	70	100	20	10	20	30
**h8**	50	30	70	70	50	60	50
	100	40	80	100	80	95	90

Katiyar et al. developed a new series of trisubstituted pyrimidine derivatives and evaluated its in vitro topoisomerase II inhibitory activity against filarial parasite *Setaria cervi*. Compounds (**h9**–**h15**) have shown 60–80% inhibition at 40 and 20 µg/ml concentrations. Structure activity relationship of most active compounds have given clear indication that amino group and 4-aminophenyl group at position-2 are very crucial in exerting topoisomerase II inhibitory activity against filarial parasite *Setaria cervi* than standard antifilarial drug (DEC) and enzyme topoisomerase II inhibitors (novobiocin, nalidixic acid) (Table 52, Fig. 14) [62].

**Table 52:** Topoisomerase II inhibitory activity against filarial parasite *Setaria cervi*

Compounds	% Inhibition at different concentrations			
	40 µg/ml	20 µg/ml	10 µg/ml	5 µg/ml
**h9**	60	60	–	–
**h10**	60	60	–	–
**h11**	80	80	80	–
**h12**	80	80	80	60
**h13**	80	80	80	60
**h14**	80	80	80	25
**h15**	70	70	70	40
DEC (antifilarial)	45	10	–	–
Novobiocin (topo II inhibitor)	80	20	10	–
Nalidixic acid (topo II inhibitor)	80	40	20	–

A new class of 2,4,6-trisubstituted bis-pyrimidines was synthesized by Parveen et al. and screened for its in vitro antiamoebic activity against HM1:IMSS strain of *Entamoeba histolytica* and toxicological studies on PC12-rat pheochoromocytoma cell line.

**Fig. 15:**
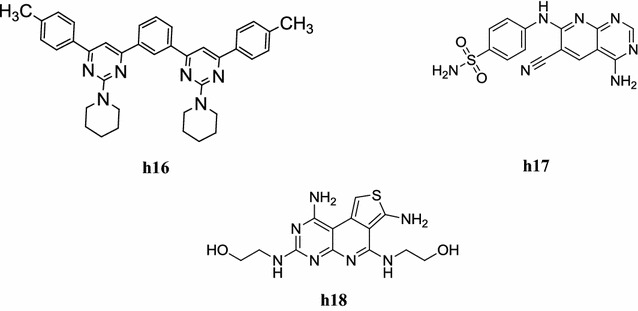
Chemical structures of the most active pyrimidine derivatives (**h16**–**h18**)

Bis-pyrimidine having methyl-substituent exhibited higher antiamoebic activity than the reference drug metronidazole (IC_50_ = 1.9 µM). Compound **h16**: 1,3-bis(2-(piperidin-1-yl)-6-(*p*-tolyl)pyrimidin-4-yl)benzene was found most active (IC_50_ = 0.10 µM) and least toxic among all the synthesized compounds (Table 53, Fig. 15) [63].

**Table 53:** Antiamoebic activity and toxicity profile of compound **h16**

Compound	Antiamoebic activity		Toxicity profile	
	(IC_50_ = µM)	SD (±)	(IC_50_ = µM)	Safety index
**h16**	0.10	0.014	> 100	> 1000
Metronidazole	9	0.020	> 100	> 52.63

*SD* standard deviation

A new class of pyrido[2,3-*d*]pyrimidine derivatives was designed and synthesized by Ibrahim and Ismail. The pyrido[2,3-*d*]pyrimidine derivatives were evaluated for their in vitro anti-proliferative activity against A431a, SNU638b, HCT116 and inhibition of CDK2-Cyclin A, CDK4/Cyclin D and EGFR enzyme. In this class, the anti-proliferative and CDK2-Cyclin A inhibitory activity of compounds, **h17** and **h18** (Fig. 15) was significantly more active than roscovotine (as standard drug) with IC_50_ values of 0.3 and 0.09 µM respectively [64].

## Conclusion

In conclusion, the biological potentials i.e. antimicrobial, anticancer, antiviral, anti-inflammatory, analgesic, antioxidant and antimalarial of pyrimidine derivatives are summarized. Pyrimidine is the important heterocyclic compound as they are being an essential constituent of cells and large number of marketed drugs. The biological activities of the pyrimidine derivatives indicated the maneuverability and versatility, which offer the medicinal chemist a continued interest in the pyrimidine skeleton in medicinal field.
